# Supplementary material for: Second harmonic generation at a time-varying interface
Source: Nat Commun. 2024 Sep 5;15:7752. doi: 10.1038/s41467-024-51588-z (PMC11377448; doi:10.1038/s41467-024-51588-z)
Supplement: Supplementary file 1 — Supplementary Information [file 41467_2024_51588_MOESM1_ESM.pdf]

Supplementary Information

Second harmonic generation at a time-varying interface

Romain Tirole[1], Stefano Vezzoli[1], Dhruv Saxena[1], Shu Yang[1], T.V. Raziman[1], Emanuele Galiffi[2], Stefan A. Maier[1,3], John B. Pendry[1], Riccardo Sapienza[1]

[1] Blackett Laboratory, Department of Physics, Imperial College London; London SW7 2BW, United Kingdom

[2] Photonics Initiative, Advanced Science Research Center, City University of New York; 85 St. Nicholas Terrace, 10031, New York, NY, USA

[3] School of Physics and Astronomy, Monash University; Clayton Victoria 3800, Australia

Corresponding authors: Romain Tirole romain.tirole16@imperial.ac.uk Riccardo Sapienza r.sapienza@imperial.ac.uk

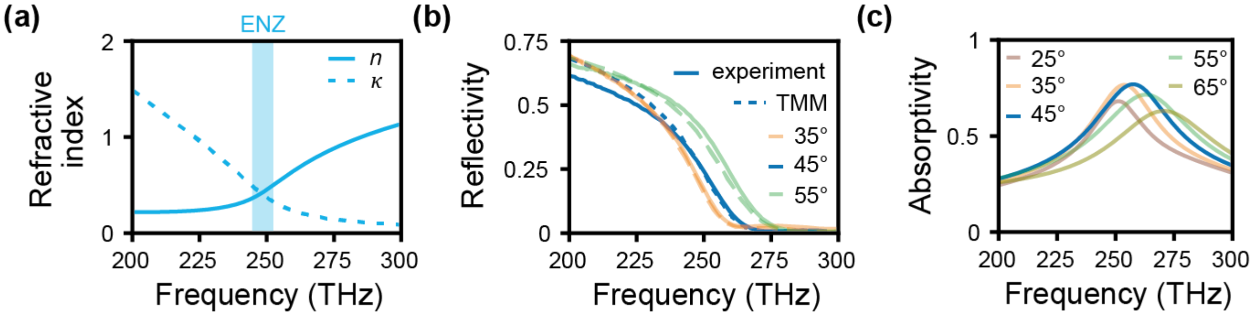

Supplementary Figure 1. Linear properties of the 310 nm ITO sample. **(a)** Refractive index of the ITO layer as a function of frequency, real (continuous) and imaginary (dashed curve). The ENZ region, centered at 248 THz, is highlighted with the shaded area. **(b)** Measured (continuous) and numerically computed with TMM (dashed) reflectivity spectrum for various incidence angles. **(c)** Corresponding simulated absorption spectra, showing a peak in absorptivity at 45 degrees, indicating the central frequency of the Berreman resonance.

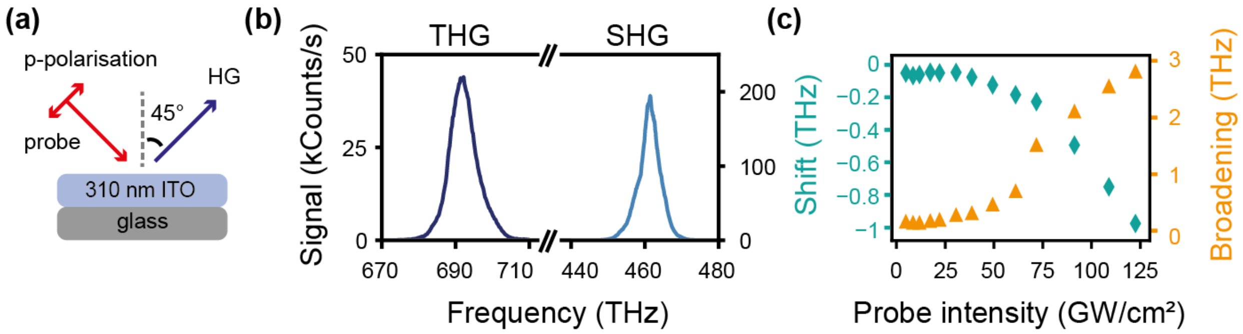

Supplementary Figure 2. Nonlinear properties of the 310 nm ITO sample. **(a)** Schematic of the harmonic generation measurement. **(b)** Third (left, dark blue curve) and second (right, light blue curve) harmonic signals for a carrier frequency of 230 THz and an illuminating intensity of 31 GW/cm^2. **(c)** Self-modulation of the probe: carrier frequency shift (cyan diamonds) and pulse bandwidth broadening (orange triangles) as a function of probe intensity, in the absence of the pump.

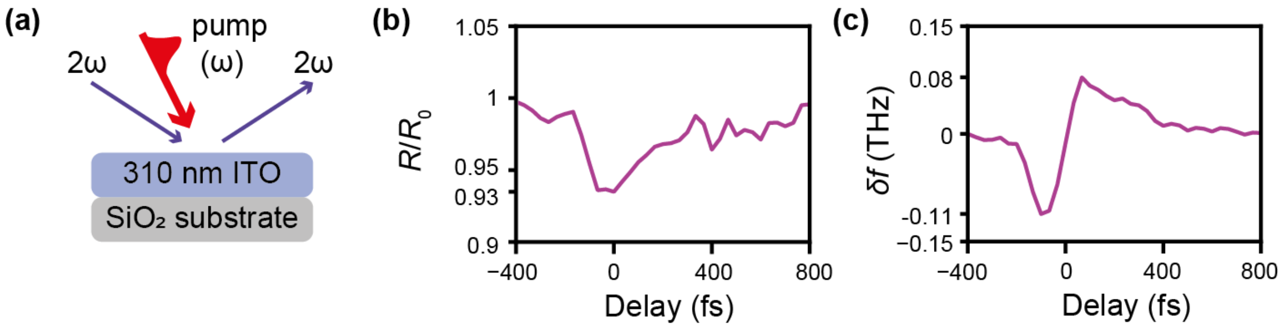

Supplementary Figure 3. Pump-probe experiment with a probe fundamental at frequency $2f =$ 460 THz. **(a)** Diagram of the pump-probe measurement with a probe centered at 460 THz. **(b)** Relative reflectivity change as a function of delay. **(c)** Frequency shift of the probe spectrum as a function of delay.

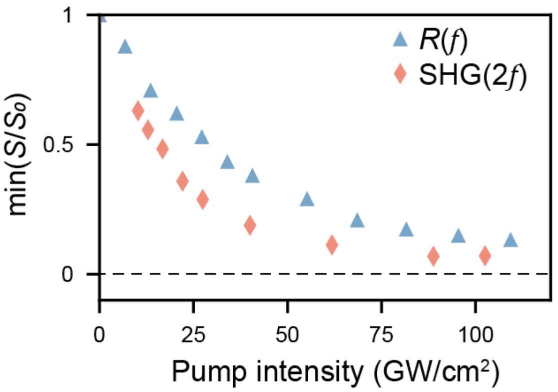

Supplementary Figure 4. Comparison of the modulation amplitude of fundamental and harmonic signals. Experimental pump intensity dependence of the maximal relative change in SHG (red diamonds) and reflected fundamental at f (blue triangles) as plotted in Fig. 2(c), on a linear scale.

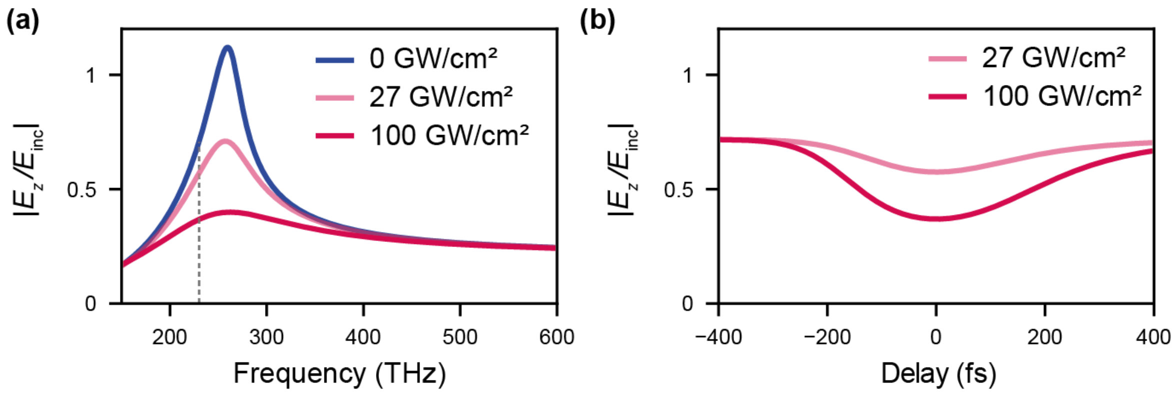

Supplementary Figure 5. Surface field at the air/ITO interface. **(a)** Coupling spectrum of the probe electric field to the z-polarised field at the surface, in the absence of pumping (blue curve) and for pump intensities of 27 GW/cm^2 and 100 GW/cm^2. The dashed grey line indicates the original carrier frequency of the probe. **(b)** Simulated surface field amplitude as a function of delay for a pump intensity of 27 GW/cm^2 (pink curve) and 100 GW/cm^2 (dark curve) and a probe carrier frequency of 230 THz.

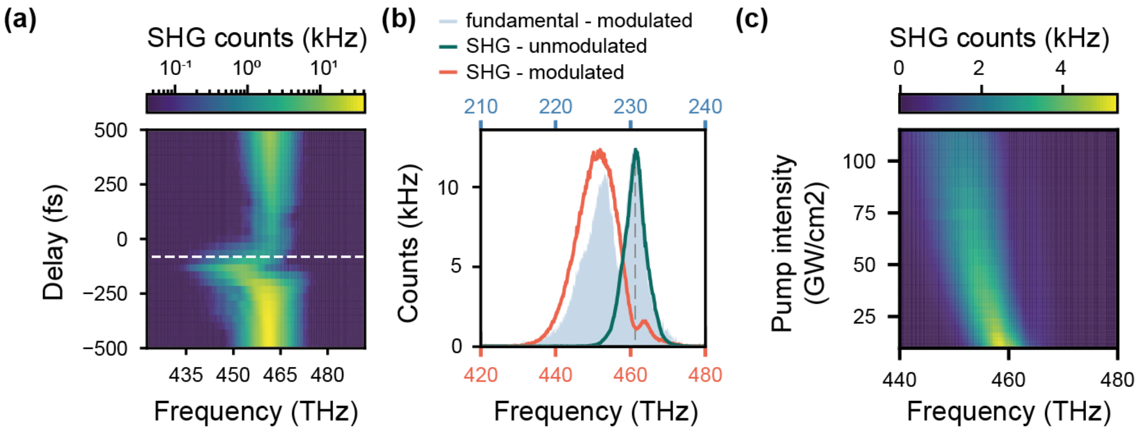

Supplementary Figure 6. Spectral modulation of the fundamental and harmonic signals. **(a)** Logarithmic scale SHG intensity spectra as a function of delay as shown in Fig. 3(a), for a probe duration of 225 fs and a pump intensity of 100 GW/cm^2. The white dashed line indicates the delay at which the spectrum in panel (b) is shown. **(b)** Comparison between the modulated fundamental (blue-shaded area) and SHG (red curve) spectra for a delay of -66 fs. The unmodulated SHG spectrum is shown in dark green for reference. The grey dashed line indicates the carrier frequency of the unmodulated fundamental/second harmonic. **(c)** Evolution of the SHG spectrum as a function of pump intensity, for a delay of -66 fs. A convergence of the frequency shift, indicating a saturation of the process, happens at ~ 50 GW/cm^2.

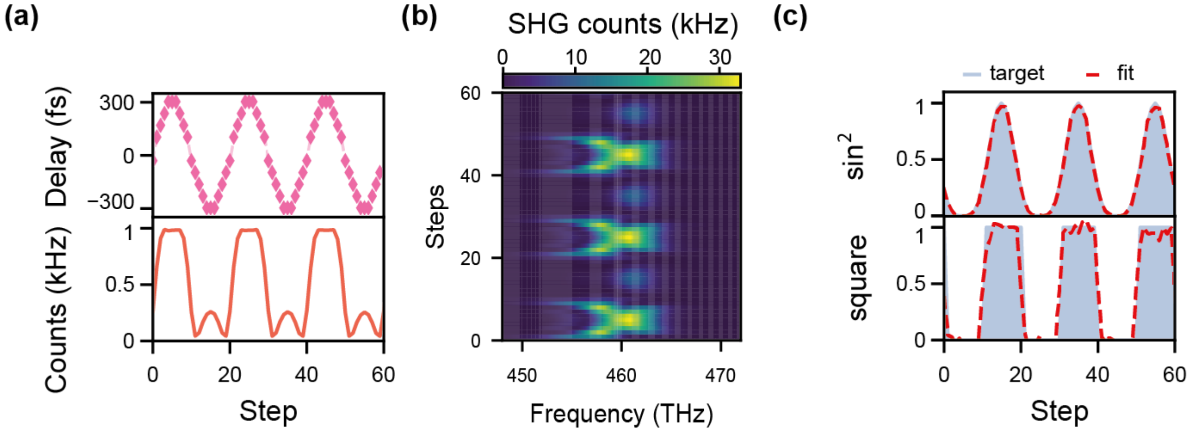

Supplementary Figure 7. Ridge regression on analogue time-modulated data. **(a)** Input of the regression algorithm: the delay steps are varied in a triangular pattern (pink curve, top), which leads to a modulation of the second harmonic counts with said steps (orange curve, bottom). **(b)** The interferogram of the SHG spectrum against step is recorded (as shown in Fig. 3(b)), the data is used for a ridge regression to link the step input to any **(c)** target function as a function of step (blue-shaded area). The regression (red dashed curve) shows good agreement with the target shape, with a mean square error of 1.57×10^{-4} for a $\sin^2$ shape (top, as in Fig. 3(c)) and 2.77×10^{-2} for a square shape (bottom).

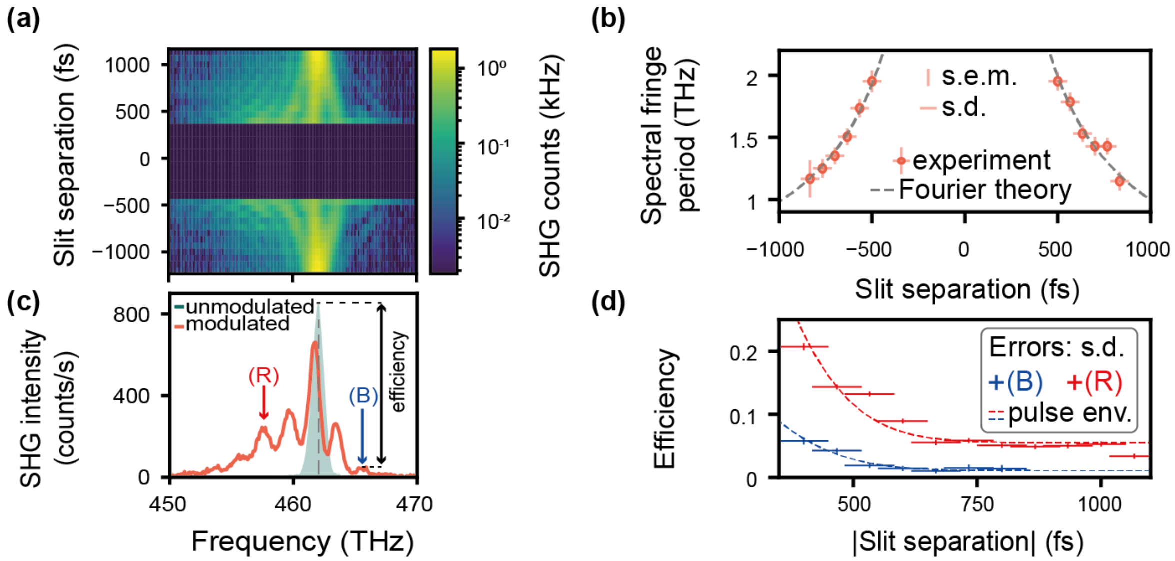

Supplementary Figure 8. A double slit experiment in time on second harmonic generated light from an ITO thin film. **(a)** SHG spectrum for various slit separations, on a logarithmic scale. Slit separations below 400 fs were not included as sum-frequency generation between two pump pulses becomes dominant. **(b)** Extracted period of the spectral oscillations against slit separation in time. The experiment (red circles) matches the inverse proportionality law from Fourier theory (dashed grey line, see Methods). The horizontal and vertical error bars represent the standard deviation (s.d.) and standard error of the mean (s.e.m.), respectively. **(c)** SHG diffraction spectrum for two slits in time separated by 467 fs as shown in Fig. 3(f), for the same pump intensity of 33 GW/cm^2. The original probe pulse, centered at 462 THz with a width of 1.12 THz, is indicated with the green-shaded area. **(d)** Efficiency of the 2nd order red peak (R) and blue peak (B) as a function of the slit separation. Frequencies generated far from the central peak carry information on the short timescale of the modulation. The existence of a 2nd blue-shifted peak is evidence of a short component in the medium recovery time. Errors are expressed as standard deviations. The trend is well fitted by the envelope of the probe Gaussian pulse (691 fs FWHM, dashed lines).

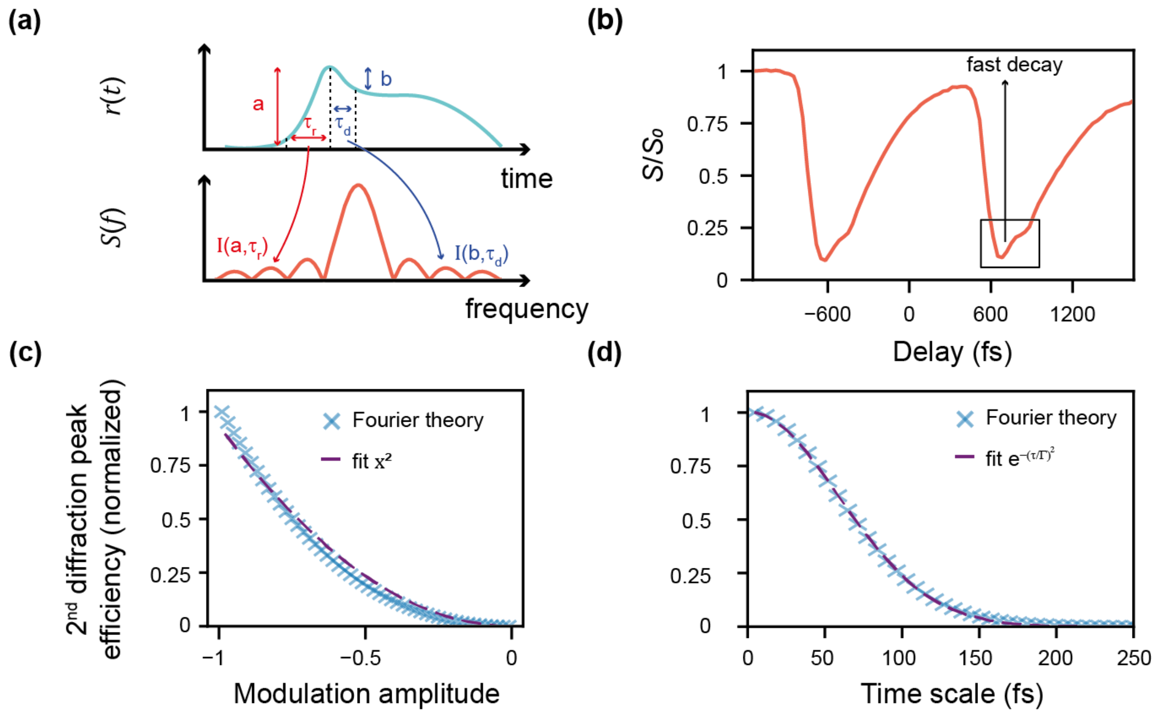

Supplementary Figure 9. Generated frequencies' dependence on dynamics and amplitude of changes in the medium. **(a)** Sketch of the respective effects of the fast excitation and relaxation time scale and amplitudes of the complex reflection coefficient r(t) on the diffraction order intensities of the spectrum S(f). **(b)** Reflected SHG signal measured as a function of delay for a double slit modulation (pump power 62.5 GW/cm^2, slit separation 1200 fs), with a probe duration of 225 fs to resolve the slit's individual effect on the SHG. **(c)** Modelled 2nd order diffraction peak dependence on modulation amplitude (blue crosses). To illustrate the quadratic behavior of the curve, a fitted curve is shown in dashed purple. **(d)** Modelled dependence of the 2nd order diffraction peak intensity on the red/blue side of the spectrum on the excitation/relaxation time (blue crosses). The agreement with a Gaussian fit (dashed purple curve) is excellent.
